# The effect of antibiotics on the clinical outcomes of patients with solid cancers undergoing immune checkpoint inhibitor treatment: a retrospective study

**DOI:** 10.1186/s12885-019-6267-z

**Published:** 2019-11-12

**Authors:** Hyunho Kim, Ji Eun Lee, Sook Hee Hong, Myung Ah. Lee, Jin Hyoung Kang, In-Ho Kim

**Affiliations:** 1 Division of Medical Oncology, Department of Internal Medicine, The Catholic University of Korea, St. Vincent’s Hospital, Suwon, Republic of Korea; 2 Division of Medical Oncology, Department of Internal Medicine, The Catholic University of Korea, Seoul St. Mary’s Hospital, Seoul, Republic of Korea; 3 Department of Internal Medicine, Seoul St. Mary’s Hospital, The Catholic University of Korea College of Medicine, 222 Banpo-daero, Seocho-gu, Seoul, 137-701 Korea

**Keywords:** Immunotherapy, Antibiotics, Survival, Solid cancer, Immune checkpoint inhibitors, Gut microbiota, Retrospective study, Korea

## Abstract

**Background:**

This study aimed to assess the effect of antibiotics on the clinical outcomes of patients with solid cancers undergoing treatment with immune checkpoint inhibitors (ICIs).

**Methods:**

The medical records of 234 patients treated with ICIs for any type of solid cancer between February 2012 and May 2018 at the Seoul St. Mary’s Hospital were retrospectively reviewed. The data of patients who received antibiotics within 60 days before the initiation of ICI treatment were analyzed. The patients’ responses to ICI treatment and their survival were evaluated.

**Results:**

Non-small-cell lung carcinoma was the most common type of cancer. About half of the patients were treated with nivolumab (51.9%), and cephalosporin (35.2%) was the most commonly used class of antibiotics. The total objective response rate was 21%. Antibiotics use was associated with a decreased objective response (odds ratio 0.466, 95% confidence interval [CI] 0.225–0.968, *p* = 0.040). The antibiotics group exhibited shorter progression-free survival (PFS) and overall survival (OS) than the no antibiotics group (median PFS: 2 months vs. 4 months, *p* < 0.001; median OS: 5 months vs. 17 months, *p* < 0.001). In the multivariate analysis, antibiotics use was a significant predictor of patient survival (PFS: hazard ratio [HR] 1.715, 95% CI 1.264–2.326, *p* = 0.001; OS: HR 1.785, 95% CI 1.265–2.519, *p* = 0.001).

**Conclusions:**

The use of antibiotics may affect the clinical outcomes of patients with solid cancers treated with ICIs. Careful prescription of antibiotics is warranted in candidates who are scheduled for ICI treatment.

**Trial registration:**

Not applicable (retrospective study).

## Background

The success of ipilimumab, which is an anti-cytotoxic T-lymphocyte-associated protein 4 (CTLA-4) monoclonal antibody (mAb), in the treatment of advanced melanoma started a new era of immune checkpoint inhibitors (ICIs) in systemic anti-cancer treatment [1]. After ipilimumab, the anti-programmed cell death protein-1 (PD-1) mAb was developed as novel ICI; it is now widely used to treat various metastatic cancers and has shown improved survival [2, 3]. Although ICI therapy has been shown to be associated with longer survival and an extended duration of the treatment response in patients with solid cancers [4, 5], not all such patients benefit from ICIs [1–5]. Only about 20% of patients treated with ICI show long-term survival of up to 10 years, and some develop severe immune-related side effects resulting in harmful outcomes such as pneumonitis, myocarditis, or hepatitis [5–7]. Therefore, many studies on the selection of candidates for ICI treatment are being conducted worldwide. For example, it has been reported that programmed death ligand-1 (PD-L1) expression and the tumor mutation burden are predictive biomarkers for improved patient outcomes [8].

ICIs targeting the PD-1/PD-L1 axis are the most widely used ICIs in the treatment of solid cancers [2, 3, 9]. PD1/PD-L1 binding inhibits stimulatory signaling of T-cell receptors, thereby reducing their proliferation, inflammatory cytokine production, and survival [9]. Anti-PD-1 and PD-L1 mAbs restore the T-cell-mediated immune response against cancer cells by preventing PD1/PD-L1 binding. Similarly, the CTLA-4 mAb restores the T-cell-mediated anticancer immune reaction by competing with cluster of differentiation 28 (CD28) binding B7, a costimulatory molecule [9].

Considering that ICIs act on T-cell immunity, we hypothesized that antibiotics use may affect the efficacy of ICI treatment in patients with solid cancers due to the association between antibiotics and the gut microbiota. Antibiotics are commonly used in clinical practice, including in the treatment of patients with cancer. They change the composition of the gut microbiota, modulating the host immune response through the development and education of the immune system [10, 11]. Unlike the 1990s, when 60–80% of intestinal bacteria were undetectable in culture tests [12], the recent development of multi-omics techniques has allowed for a more comprehensive analysis of gut microbiota composition through deep 16S rRNA sequencing [12–15]. Using this methodology, preclinical studies showed that the use of antibiotics can change T-cell immunity by altering the gut microbiota [10–12].

This study aimed to investigate the effect of antibiotics use on the clinical outcomes of patients with solid cancers receiving ICI treatment.

## Methods

### Study population

This retrospective study was approved by the Institutional Review Board (IRB) of the Seoul St. Mary’s Hospital of the Catholic University of Korea (KC19RESI0114). The need for informed consent was waived by the IRB of the Seoul St. Mary’s Hospital of the Catholic University of Korea due to the retrospective study design.

The medical records of patients treated with ICIs (anti-PD-1, anti-PD-L1, and anti CTLA-4 mAbs) for any type of solid cancer at the hospital between February 2012 and May 2018 were reviewed. Patients who died within 4 weeks of antibiotics administration were excluded as they either had a very poor performance status or did not recover from a severe infection. The treatment regimens included ICI alone, ICI combination therapy, and ICI plus chemotherapy, regardless of previous anticancer treatment.

### Variables and outcomes

The clinicopathologic characteristics of all patients were assessed. Medical records were reviewed after classifying the patients according to the timing of antibiotics administration (no antibiotics, antibiotics use within 30 days of ICI treatment initiation, and antibiotics use 31–60 days before ICI treatment initiation). Previous studies showed that alterations in the gut microbiota occurred in less than 1 week after treatment initiation and lasted for 1–3 months up to 2 years [16–18]. Considering the estimated minimum recovery time of the gut microbiota, most patients treated with antibiotics within 1 to 2 months before the start of ICI treatments will not have a recovered gut microbiota.

We analyzed the presence of bacteremia (indicating severe systemic infection), when antibiotics treatment was initiated, the type of antibiotics used, the route of administration, and the treatment duration. As the study population was highly heterogeneous, we also performed a subgroup analysis of patients with non-small-cell lung carcinoma (NSCLC) as this was the most common type of cancer identified in this study. In patients with NSCLC, PD-L1 expression, the presence of an epidermal growth factor receptor (*EGFR*) mutation, and the histological subtype were also assessed.

To evaluate the treatment response, we reviewed the results of imaging studies including computed tomography and magnetic resonance imaging. Radiological changes were evaluated using the Response Evaluation Criteria for Solid Tumors, version 1.1 [19]. An objective response was categorized as a complete response (CR) or partial response (PR), while disease control was categorized as CR, PR, or stable disease (SD). All patients were followed up until death or data lock (January 10, 2019).

### Statistical analysis

Patients were categorized according to the status of antibiotic use (yes vs. no) within 60 days prior to the start of ICI treatment. The patients’ baseline characteristics were compared using the Chi-squared or Fisher’s exact test for categorical variables. Survival curves were calculated using the Kaplan-Meier method, and the log-rank test was used to compare the survival curves. A Cox proportional hazards model was used to perform a multivariate analysis to assess prognostic variables for progression-free survival (PFS) and overall survival (OS). The Chi-squared test was employed to determine differences in the overall response between the antibiotics and no antibiotics groups; several therapeutic windows were evaluated (no antibiotics, antibiotics use within 30 days of ICI treatment initiation, and antibiotics use 31–60 days before ICI treatment initiation). The same analyses were performed in the NSCLC subgroup.

All statistical analyses were performed using the SPSS software (version 24; IBM corp., Armonk, NY, USA). A two-sided *p*-value < 0.05 was considered significant.

## Results

### Baseline characteristics of the patients

A total of 234 patients were included in the study. Table 1 shows the patients’ characteristics by antibiotic use. NSCLC was the most common type of cancer. The most common treatment regimen used was ICI alone (*N* = 189, 80.8%). ICI combination therapy (*N* = 20, 8.5%) consisted mostly of nivolumab with ipilimumab. Of all patients, 108 (46.2%) received antibiotics at least once within 60 days prior to the initiation of ICI treatment. Cephalosporin was the most commonly used antibiotic (*N* = 38, 35.2%), followed by quinolone (*N* = 26, 24.1%). Oral antibiotics were more commonly prescribed than intravenous antibiotics (62% vs. 38%). Most patients received antibiotics for prophylactic use (*N* = 79, 73.1%); accordingly, only 26.9% of the patients (*N* = 29) were administered for treatment. Anti-fungal agents were used in only one patient who was treated with oral fluconazole due to oral candidiasis. The antibiotics group had a higher proportion of patients with a high Eastern Cooperative Oncology Group performance status (ECOG PS) of 2–3.

**Table 1:** Baseline characteristics (*N* = 234)

	Total (%)	No Antibiotics (%)	Antibiotics (%)	*p* value
Age				
< 65	110 (47)	56 (44.4)	54 (50)	0.396
≥ 65	124 (53)	70 (55.6)	54 (50)	
Sex				
Male	168 (71.8)	91 (72.2)	77 (71.8)	0.875
Female	66 (28.2)	35 (27.8)	31 (28.2)	
ECOG score				
0–1	200 (87.7)	112 (92.6)	88 (82.2)	0.018
2–3	28 (12.3)	9 (7.4)	19 (17.8)	
unknown	6	5	1	
Diagnosis				
NSCLC	131 (56)	71 (56.3)	48 (55.6)	0.903
Others^a^	103 (44)	55 (43.7)	60 (44.4)	
Stage				
III	9 (3.8)	6 (4.8)	3 (2.8)	0.511
IV	225 (96.2)	120 (95.2)	105 (97.2)	
Number of metastatic organ				
0 or 1	151 (64.5)	78 (61.9)	73 (67.6)	0.365
≥ 2	83 (35.5)	48 (38.1)	35 (32.4)	
Number of treatment line				
1st	72 (30.8)	45 (35.7)	27 (25)	0.198
2nd	96 (41)	49 (38.9)	47 (43.5)	
≥ 3rd	66 (28.2)	32 (25.4)	34 (31.5)	
ICI				
Nivolumab	135 (57.7)	79 (62.7)	56 (51.9)	0.242
Pembrolizumab	62 (26.5)	29 (23)	33 (30.6)	
Others^b^	37 (15.8)	18 (14.3)	19 (17.6)	
Treatment combination				
ICI alone	189 (80.8)	97 (77)	92 (85.2)	0.063
ICI with ICI	20 (8.5)	10 (7.9)	10 (9.3)	
ICI with chemotherapy	25 (10.7)	19 (15.1)	6 (5.6)	
Clinical trial				
Yes	108 (46.2)	72 (57.1)	36 (33.3)	< 0.001
No	126 (53.8)	54 (42.9)	72 (66.7)	
Antibiotics type				
No Antibiotics	126 (53.8)	126		
Antibiotics	108 (46.2)	Cephalosporins	38 (35.2)	
		Fluoroquinolones	26 (24.1)	
		Beta-lactam/Betalactamase inhibitors	18 (16.6)	
		Others^c^	26 (24.1)	
Administration Route				
Oral	67		67 (62)	
Intravenous	41		41 (38)	

^a^Melanoma, *N* = 27; Bladder, *N* = 8; Renal cell carcinoma, *N* = 9, Head and Neck cancer, *N* = 16; Stomach cancer, *N* = 21; Hepato cellular carcinoma, *N* = 7; Esophageal cancer, *N* = 5; Small cell lung cancer, *N* = 3; Anal cancer, Cervical cancer, Colorectal cancer, Jejunal cancer, MUO, Ovarian cancer, Sarcoma, *N* = 1, resepcetively

^b^Avelumab, *N* = 9; Durvalumab, *N* = 5; Atezoliaumab, *N* = 4; Ipilimumab, *N* = 15

^c^Carbapenem, Glycopeptides, Macrolides and etc

### Survival and response to treatment

**Fig. 1:**
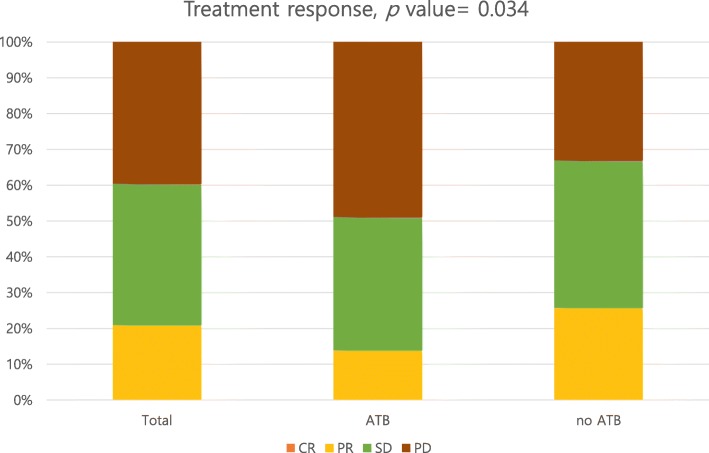
Immune checkpoint inhibitors; treatment response in solid cancer

**Fig. 2:**
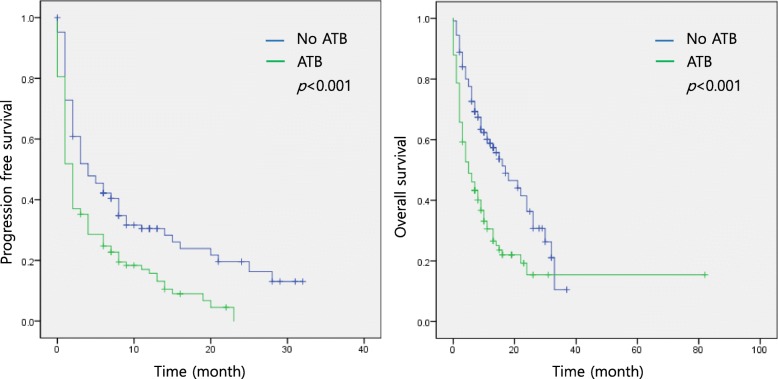
Survival curves and the impact of antibiotics in solid cancer patients treated with ICIs. ATB: antibiotics

The patients’ responses to treatment are described in Fig. 1 and Table 2. None of the patients achieved a CR. The total objective response rate was 21%. A history of antibiotics use was associated with a decreased objective response (odds ratio [OR] 0.466, 95% confidence interval [CI] 0.225–0.968; *p* = 0.040) and decreased disease control (OR 0.517, 95% CI 0.294–0.910; *p* = 0.022). The antibiotics group showed shorter PFS and OS than the no antibiotics group (median PFS: 2 months vs. 4 months, *p* < 0.001; median OS: 5 months vs. 17 months, *p* < 0.001) (Fig. 2).

**Table 2:** Immune check point inhibitors, Treatment response in solid cancer

	Total	ATB	no ATB	*p* value		Total	ATB	no ATB	*p* value
CR	0	0	0	0.034	OR	44 (21%)	12 (14%)	32 (26%)	0.038
PR	44 (21%)	12 (14%)	32 (26%)		nOR	166 (79%)	74 (86%)	92 (74%)	
SD	83 (39.5%)	32 (37%)	51 (41%)		DC	127 (60%)	44 (51%)	83 (67%)	0.022
PD	83 (39.5%)	42 (49%)	41 (33%)		nDC	83 (40%)	42 (49%)	41 (33%)	
Total	210	86	124		Total	210	86	124	

Non-evaluated, *N* = 24

*ATB* Antibiotics

In the univariate analysis, antibiotics use within 60 days before the initiation of ICI treatment, the ECOG PS, the number of metastatic organs, cancer stage, previous chemotherapy, combination therapy, participation in a clinical trial, and antibiotics administration during ICI treatment affected both OS and PFS (Table 3). In the multivariate analysis, a history of antibiotics use within 60 days prior to the start of ICI therapy was significantly associated with survival (PFS: hazard ratio [HR] 1.715, 95% CI 1.264–2.326, *p* = 0.001; OS: HR 1.785, 95% CI 1.265–2.519, *p* = 0.001) (Table 3).

**Table 3:** Multivariate analysis

	PFS			OS		
	Multivariate			Multivariate		
	HR	95% CI	*p* value	HR	95% CI	*p* value
ECOG						
0 or 1			0.003			< 0.001
2 or 3	1.907	1.245–2.921		2.607	1.666–4.080	
Diagnosis						
NSCLC			0.062			
Others^a^	1.328	0.986–1.788				
Stage						
III			0.025			0.103
IV	2.605	1.130–6.004		2.332	0.842–6.461	
Number of metastatic organ						
0 or 1						0.007
≥ 2				1.6	1.135–2.256	
Number of treatment line						
1st			< 0.001			
2nd	2.035	1.410–2.939	< 0.001			
≥ 3rd	1.885	1.269–2.800	0.002			
Clinical trial						
Yes						0.001
No				1.829	1.266–2.641	
Antibiotics during ICI						
No			0.037			
Yes	0.7	0.501–0.978				
Antibiotics before ICI						
No			0.001			0.001
Yes	1.715	1.264–2.326		1.785	1.265–2.519	

^a^Melanoma, Bladder cancer, Renal cell carcinoma, Head and Neck cancer, Stomach cancer, Hepato cellular carcinoma, Esophageal cancer, Small cell lung cancer, Anal cancer, Cervical cancer, Colorectal cancer, Jejunal cancer, MUO, Ovarian cancer, Sarcoma

We then classified the study population into patients who received no antibiotics, those who received antibiotics within 30 days before ICI therapy initiation, and those who received antibiotics 31–60 days before ICI therapy and conducted the same analyses. A history of antibiotics use negatively affected the treatment response (rate of progressive disease [PD]: none vs. 30 days vs. 60 days: 33.1% vs. 43.6% vs. 53.2%; *p* = 0.013) (Additional file 1). Patients receiving antibiotics had shorter PFS and OS than those not receiving antibiotics (none vs. 30 days vs. 60 days: median PFS: 4 months vs. 1 months vs. 2 months, *p* < 0.001; median OS: 17 months vs. 4 months vs. 7 months, *p* < 0.001) (Additional file 2). In the multivariate analysis, a history of antibiotics use was an independent prognostic factor (PFS, *p* = 0.002; OS, *p* < 0.001) (Additional file 3).

### NSCLC subgroup: survival and objective response

The baseline characteristics of the NSCLC subgroup are shown in Table 4. Of all patients, 131 (56%) had NSCLC; of these, 60 (45.8%) received antibiotics within 60 days prior to ICI therapy initiation. The most common class of antibiotics was cephalosporin; oral antibiotics were more frequently prescribed than intravenous antibiotics. We found similar rates of brain metastasis, previous chemotherapy, the histologic type of NSCLC, PD-L1 expression, and the presence of an *EGFR* mutation in the antibiotics and no antibiotics group. The antibiotics group had higher proportions of patients with an ECOG PS of 2–3 and those enrolled in clinical trials when compared to the no antibiotics group.

**Table 4:** Baseline charateristics in NSCLC (*N* = 131)

	Total (%)	No Antibiotics (%)	Antibiotics (%)	*p* value
Age				
< 65	56 (42.7)	28 (39.4)	28 (46.7)	0.405
≥ 65	75 (57.3)	43 (60.6)	32 (53.3)	
Sex				
Male	99 (75.6)	54 (76.1)	45 (75)	0.889
Female	32 (24.4)	17 (23.9)	15 (25)	
ECOG				
0–1	116 (89.9)	66 (95.7)	50 (83.3)	0.02
2–3	13 (10.1)	3 (4.3)	10 (16.7)	
Unkown	2	2	0	
Stage				
III	4 (3.1)	3 (4.2)	1 (1.7)	0.625
IV	127 (96.9)	68 (95.8)	59 (98.3)	
Number of metastatic organ				
0 or 1	85 (64.9)	47 (66.2)	38 (63.3)	0.732
≥ 2	46 (35.1)	24 (33.8)	22 (36.7)	
Brain metastasis				
No	106 (80.9)	57 (80.3)	49 (81.7)	0.841
Yes	25 (19.1)	14 (19.7)	11 (18.3)	
Number of treatment line				
1st	39 (29.8)	25 (35.2)	14 (23.3)	0.304
2nd	56 (42.7)	29 (40.8)	27 (45)	
≥ 3rd	36 (27.5)	17 (23.9)	19 (31.7)	
ICI				
Nivolumab	71 (54.2)	44 (62)	27 (45)	0.024
Pembrolizumab	41 (31.3)	15 (21.1)	26 (43.3)	
Others^a^	19 (14.5)	12 (16.9)	7 (11.7)	
Treatment combination				
ICI alone	104 (79.4)	53 (74.6)	51 (85)	0.117
ICI with ICI	7 (5.3)	3 (4.2)	4 (6.7)	
ICI with chemotherapy	20 (15.3)	15 (21.1)	5 (8.3)	
Clinical trial				
Yes	65 (49.6)	46 (64.8)	19 (31.7)	< 0.001
No	66 (50.4)	25 (35.2)	41 (68.3)	
Hisotologic subtype				
Adenocarcinoma	83 (63.4)	46 (64.8)	37 (61.7)	
Squamous cell carcinoma	44 (33.6)	24 (33.8)	20 (33.3)	
Others^b^	4 (3.1)	1 (1.4)	3 (5)	
PD-L1				
Negative	14 (13.6)	11 (20.4)	3 (6.1)	0.058
Low	30 (29.1)	17 (31.5)	13 (26.5)	
High	59 (57.3)	26 (48.1)	33 (67.3)	
Unkown	28	17	11	
EGFR				
Negative	92 (88.5)	53 (89.8)	39 (86.7)	0.617
Positive	12 (11.5)	6 (10.2)	6 (13.3)	
Unkown	27	12	15	
Antibiotics type				
No Antibiotics	71 (54.2)			
Antibiotics	60 (45.8)	Cephalosporins	17 (28.3)	
		Fluoroquinolones	16 (26.7)	
		Beta-lactam/Betalactamase inhibitors	9 (15)	
		Others^c^	18 (30)	
Administration Route				
Oral	37		37 (61.7)	
Intravenous	23		23 (38.3)	

^a^Avelumab, *N* = 6; Durvalumab, *N* = 5; Ipilimumab, *N* = 8

^b^Sarcomatoid carcinoma, *N* = 2, Large cell neuroendocrine carcinoma, *N* = 1; Poorly differentiated carcinoma, *N* = 1

^c^Carbapenem, Glycopeptides, Macrolides and etc.

**Fig. 3:**
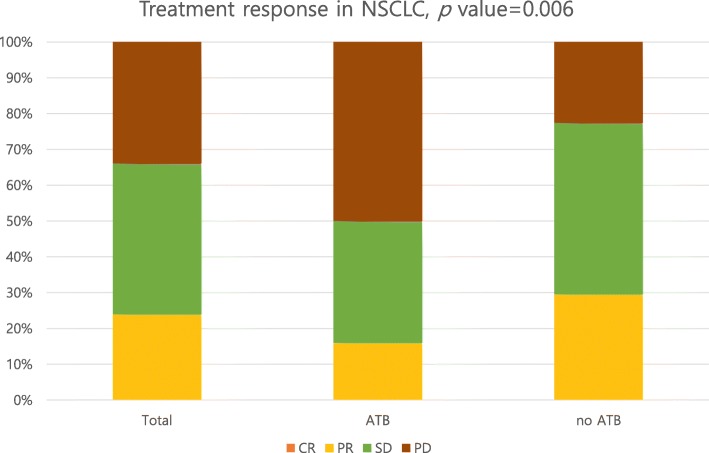
Immune checkpoint inhibitors; treatment response in NSCLC

**Fig. 4:**
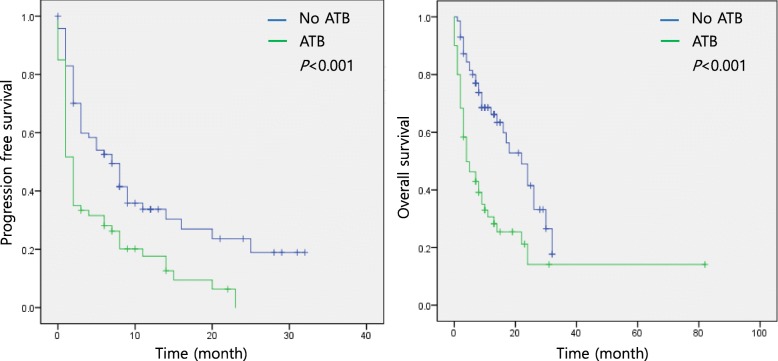
Survival curves and the impact of antibiotics in NSCLC patients treated with ICIs. ATB: antibiotics

A history of antibiotics use was associated with a higher rate of PD (antibiotics vs. no antibiotics: 50% vs. 22.5%, *p* = 0.006) and a decreased treatment response; however, there was no statistically significant difference in the objective response rate between the two groups (antibiotics vs. no antibiotics: objective response rate: 16% vs. 29.6%, *p* = 0.085; disease control rate: 50% vs. 77.5%, *p* = 0.002) (Fig. 3 and Table 5). The antibiotics group exhibited shorter PFS and OS than the no antibiotics group (median PFS: 2 months vs. 7 months, *p* < 0.001; median OS: 4 months vs. 22 months, *p* < 0.001) (Fig. 4). The multivariate analysis revealed that a history of antibiotics use, the ECOG PS, cancer stage, number of metastatic organs, brain metastasis, participation in a clinical trial, PD-L1 expression, and the presence of an *EGFR* mutation were independent predictors of survival (PFS: HR 2.379, 95% CI 1.281–4.418, *p* = 0.006; OS: HR 3.834, 95% CI 1.736–8.469, *p* = 0.001) (Table 6). Both PFS and OS were significantly different between patients not receiving antibiotics and those who underwent antibiotics treatment within 30 days or within 31–60 days prior to ICI therapy initiation (no antibiotics vs. 30 days vs. 31–60 days: median PFS: 7 months vs. 1 month vs. 2 months, *p* = 0.001; median OS: 22 months vs. 4 months vs. 8 months, *p* < 0.001) (Additional file 4).

**Table 5:** Immune checkpoint inhibitors, Treatment response in NSCLC

	Total	ATB	no ATB	*p* value		Total	ATB	no ATB	*p* value
CR	0	0	0	0.006	OR	29 (24%)	8 (16%)	21 (30%)	0.085
PR	29 (24%)	8 (16%)	21 (30%)		nOR	92 (76%)	42 (84%)	50 (70%)	
SD	51 (42%)	17 (34%)	34 (48%)		DC	80 (66%)	25 (50%)	55 (78%)	0.002
PD	41 (34%)	25 (50%)	16 (22%)		nDC	41 (34%)	25 (50%)	16 (22%)	
Total	121	50	71		Total	121	50	71	

Non-evaluated, *N* = 10

*ATB* Antibiotics

**Table 6:** Multivariate analysis in NSCLC

	PFS						OS					
	Univariate			Multivariate			Univariate			Multivariate		
	HR	95% CI	*p* value	HR	95% CI	*p* value	HR	95% CI	*p* value	HR	95% CI	*p* value
ECOG												
0 or 1	1.0		0.005	1.0		0.003	1.0		0.006	1.0		0.015
2 or 3	2.316	1.281–4.187		3.945	1.573–9.896		2.464	1.288–4.711		3.894	1.301–11.660	
ICI												
Nivolumab	1.0		0.891				1.0		0.363	1.0		0.039
Pembrolizumab	1.115	0.716–1.736	0.631				1.31	0.790–2.173	0.295	3.342	1.187–9.411	0.022
Others	1.043	0.595–1.828	0.884				0.801	0.410–1.564	0.516	2.651	0.676–10.403	0.162
Stage												
III	1.0		0.101	1.0		0.141	1.0		0.216	1.0		0.127
IV	3.241	0.794–13.223		4.645	0.601–35.859		2.439	0.593–10.030		5.747	0.610–54.146	
Number of metastatic organ												
0 or 1	1.0		0.007	1.0		0.078	1.0		< 0.001	1.0		0.014
≥ 2	1.754	1.170–2.630		1.681	0.943–2.996		2.732	1.697–4.397		2.401	1.193–4.830	
Brain metastasis												
No	1.0		0.502	1.0		0.026	1.0		0.979	1.0		0.112
Yes	0.832	0.485–1.425		0.373	0.157–0.890		1.008	0.553–1.836		0.398	0.128–1.241	
Number of treatment line												
1st	1.0		0.064				1.0		0.324			
2nd	1.484	0.911–2.418	0.113				1.245	0.717–2.163	0.437			
≥ 3rd	1.855	1.102–3.121	0.02				1.568	0.871–2.822	0.134			
Clinical trial												
Yes	1.0		0.032	1.0		0.011	1.0		0.018	1.0		0.031
No	1.554	1.039–2.324		2.35	1.217–4.537		1.782	1.104–2.877		3.27	1.116–9.584	
Histologic subtype												
Adenocarcinoma	1.0		0.768				1.0		0.855			
Squamous cell carcinoma	1.064	0.703–1.610					0.956	0.59.-1.542				
PD-L1												
Negative			0.226	1.0		0.064	1.0		0.396	1.0		0.024
Low	1.723	0.776–3.827	0.181	0.952	0.372–2.440	0.919	1.839	0.676–4.999	0.233	0.802	0.229–2.817	0.731
High	1.165	0.546–2.486	0.693	0.447	0.179–1.117	0.085	1.329	0.515–3.433	0.557	0.218	0.055–0.866	0.030
EGFR												
Negative	1.0		0.196	1.0		0.061	1.0		0.376	1.0		0.072
Positive	1.554	0.796–3.031		2.574	0.956–6.929		1.401	0.664–2.956		2.964	0.906–9.695	
Antibiotics before ICI												
No	1.0		0.001	1.0		0.006	1.0		< 0.001	1.0		0.001
Yes	1.948	1.310–2.898		2.379	1.281–4.418		2.476	1.568–3.911		3.834	1.736–8.469	

### Survival outcomes by type of antibiotics and route of administration

We examined the patients’ survival curves according to the type of antibiotics used and found no significant differences in survival in both, all patients (PFS: *p* = 0.072; OS: *p* = 0.508) and those with NSCLC (PFS: *p* = 0.111; OS: *p* = 0.694).

Among all patients, we found no statistically significant differences in median PFS and OS by type of antibiotics (cephalosporins vs. quinolones vs. beta-lactam/beta-lactamase inhibitors (BLBLIs) vs. others: median PFS: 2 months vs. 1 months vs. 1 months vs. 2 months; median OS: 5 month vs. 4 month vs. 6 months vs. 7 months). In the NSCLC group, patients treated with a BLBLI showed trends of longer PFS and OS when compared to those treated with other types of antibiotics (cephalosporins vs. quinolones vs. BLBLI vs. others: median PFS: 1 months vs. 1 months vs. 8 months vs. 2 months; median OS: 3 month vs. 4 month vs. 9 months vs. 7 months); however, the differences were not statistically significant.

All nine patients in the NSCLC subgroup treated with a BLBLI received antibiotics via the intravenous route. We hypothesized that the route of antibiotics administration may affect survival. However, there was no significant difference in survival between patients receiving oral agents and those receiving intravenous agents (PFS: *p* = 0.232; OS: *p* = 0.531). Moreover, the administration of antibiotics during ICI therapy was not associated with survival (PFS: *p* = 0.084; OS: *p* = 0.845).

### Survival outcomes by duration of antibiotics treatment

Last, we examined the effect of the duration of antibiotics use on patient survival. Among 108 patients who received antibiotics, 25 were treated with antibiotics < 7 days. These patients exhibited poorer survival but did not show a statistically significant difference in median PFS when compared to patients receiving no antibiotics (median PFS: 4 months in both groups, *p* = 0.077; median OS: 10 months vs. 17 months, *p* = 0.032) (Additional file 5). Patients undergoing antibiotics treatment for > 7 days exhibited statistically significant shorter PFS and OS than those not undergoing antibiotics treatment (median PFS: 1 month vs. 4 months, *p* < 0.001; median OS: 4 months vs. 14 months, *p* < 0.001).

## Discussion

In this study, we analyzed the effect of antibiotics use on clinical outcomes in patients with solid cancers undergoing treatment with ICIs. Almost half of the patients (46.2%) received antibiotics prior to the start of ICI therapy. A history of antibiotics use showed a significant association with ICI treatment outcomes and survival; similar results were seen in the NSCLC subgroup.

When interpreting our results, several issues should be considered. First, patients treated with antibiotics had a poorer general condition (as measured by the ECOG PS) when compared to those not receiving antibiotics. The proportion of patients with an ECOG PS of 2–3 was significantly lower in the no antibiotics group than in the antibiotics groups (7.4% vs. 17.8%). As expected, we found a significant difference in median OS between the low and high ECOG PS subgroups (11 months vs. 2 months, *p* < 0.001). However, the total proportion of patients with an ECOG PS of 2–3 was small at 11.9% (specifically, only 4 patients [1.7%] had an ECOG PS of 3); thus, the majority of patients analyzed had a good performance status. Moreover, the shapes of the ECOG PS survival curves were different between the antibiotics groups at the end of the curves (Additional file 6). In the multivariate analysis, when controlling for the ECOG PS, a history of antibiotics use was an independent prognostic factor. Furthermore, the most common reason for antibiotics use was prophylaxis (79 patients, 73.1%) which was defined as the response to an elevated C-reactive protein level only (without fever or specific localized symptoms); bacteremia was observed in only 4 of 108 patients (3.7%) who were treated with antibiotics. In other words, we presume that severe systemic infection and a poor performance status had a limited effect on the association between antibiotics use and ICI treatment-related outcomes in this study, although the ECOG PS is a well-known prognostic factor.

Our data revealed a higher rate of PD and lower objective response rate in the antibiotics group than in the non-antibiotics group (PD: 49% vs. 33%; objective response rate: 18% vs. 26%). Meanwhile, the antibiotics group had shorter PFS than the no antibiotics group (2 months vs. 4 months). These findings suggest that the use of antibiotics can have a negative effect on the efficacy of ICI treatment. Previous studies support the possibility that antibiotics administration affects the clinical efficacy of ICI [16, 20]. Derosa et al. reported an increased risk of PD (75% vs. 22%, *p* < 0.01) as well as shorter PFS and OS in patients with renal cell carcinoma or NSCLC treated with antibiotics [16]. Similarly, Ahmed et al. showed that patients with various types of solid cancers receiving broad-spectrum antibiotics had a lower response rate (25% vs. 61%, *p* = 0.02) and shorter PFS than those not receiving antibiotics [20]. These data indicate that changes in the intestinal flora due to the effects of antibiotics may be one of the causes of the poor efficacy of ICI.

Trillions of bacteria live along the gastrointestinal tract [11]. Under normal conditions, the host immune system maintains beneficial strains and prevents the over-proliferation and rapid growth of non-beneficial strains [10]. Exposure to antibiotics can impair the homeostasis of gut microbiota, resulting in decreased microbial diversity (the variability of harmful and healthy bacteria) [12]. Previous studies reported that cephalosporins and BLBLI, which were the most common antibiotics used in this study, modulated the composition of *Firmicutes*, *Bacteroidetes*, and *Proteobacteria* in the intestinal-bacterial community [12, 21]. Fluoroquinolone was also shown to play an important role in modulating the gut microbiota, with the degree of alterations differing according to the category of quinolones used [12, 22]. The disruption of the gut microbiota affects systemic T-cell activity and their number, along with an impairment of dendritic cell migration, immunoglobulin levels, and interferon-gamma levels [10]. Abt et al. showed that exposure to antibiotics was associated with a reduced expansion of lymphocytic choriomeningitis virus (LCMV)-specific CD8+ T cells in mice, releasing effector molecules such as interleukin-2 and interferon-gamma [23]. Considering these previous studies, a well-designed prospective study using stool samples is needed to confirm how antibiotics change the gut microbiota, ultimately causing altered ICI efficacy.

The type of antibiotics, route of administration, and duration of antibiotics treatment were not associated with treatment outcomes in our study. Arboleya et al. reported that beta-lactams and BLBLI reduced the proportion of *Actinobacteria*, including *Bifidobacterium,* in preterm infants [24]. In another study, ciprofloxacin was associated with a decreased proportion of *Bifidobacterium* [11, 25]. Although previous studies reported that both BLBLI and ciprofloxacin decreased intraluminal *Bifidobacterium*, the specific strain linked to the efficacy of ICI and how the type of antibiotics affects the clinical outcomes of patients treated with ICIs remain unclear. We considered that the intra-luminal concentration of antibiotics differs according to the route of administration. Our findings showed that the ratio between oral and intravenous antibiotic use was highly unbalanced. For example, fluoroquinolones, including ciprofloxacin with a bioavailability of about 70% in the oral route [26], was orally administered in only 1 of the 26 patients. Thus, we could not adequately compare oral and intravenous use. In terms of the period of antibiotics use, the most common antibiotics treatment duration was ≥7 days (82 patients, 76%). Short-term antibiotics use can also affect the gut microbiota [11, 17], and our study population included patients who received antibiotics for < 7 days. Unlike the use of antibiotics before ICI therapy, antibiotics use during ICI therapy did not affect survival in this study. This may be because ICI not only reactivates cytotoxic T cells but also modulates memory T cells [27]. Modified T-cell immunity caused by the first administration of ICI may persist thereafter and Survival may therefore not be significantly affected by antibiotics use during ICI therapy.

This study had some limitations. As discussed earlier, a higher proportion of patients treated with antibiotics had a poor performance status when compared to those who did not receive antibiotics; the ECOG PS is an important prognostic factor in itself. ICI treatment can be continued beyond progression as long as patients show no significant deterioration, which can affect the evaluation of progression. Thus, caution must be exercised when interpreting our data. Second, the study design was a retrospective review of medical records. Therefore, we could not perform culture testing of the patients’ stool samples and utilize multi-omics technologies to confirm gut microbiota alterations according to antibiotics administration. Accordingly, we were unable to analyze if differences in the gut microbiota affected ICI treatment outcomes. In a previous study, an abundance of *Akkermansia muciniphila* was correlated with the anti-PD-1 immunotherapy response in patients who underwent a stool metagenomics analysis prior to treatment [28]. Sivan et al. reported that the oral administration of *Bifidobacterium* enhanced the response of anti-PD-1 therapy in mice with melanoma [29]. Vetizou et al. showed that *Bacteroides* species modulated the efficacy of anti-CTLA-4 therapy in mice treated with antibiotics [30]. Considering these and our findings, fecal microbiota transplantation (FMT) may ameliorate ICI treatment outcomes in patients with solid cancers. Routy et al. showed that FMT from ICI responders into germ-free or antibiotic-treated mice improved the tumor control of anti PD-1 mAbs, whereas FMT from non-responders was unable to achieve tumor control [28]. Oral administration of *A. muciniphila* with FMT of non-responder feces restored the antitumor effect of anti-PD-1 mAb through the accumulation of CCR9+ CXCR3+ CD4+ T lymphocytes in mouse tumor beds [28]. Third, our study population was heterogeneous as it consisted of patients who underwent treatment for various cancer types. According to the type of cancer, cancer biology and treatment course are different. Therefore, a study in patients with a homogeneous cancer type is ideal. However, the sample size of this study was small; therefore, we had to evaluate all patients treated with ICIs, irrespective of the type of cancer. Last, this study was designed without controlling for host factors related to the gut microbiota such as lifestyle and the neonatal environment [12]. Hence, further studies in homogeneous patient groups are needed.

## Conclusion

The findings of our study suggest that the use of antibiotics may affect the clinical outcomes of patients with solid cancers treated with ICI. Prescribing antibiotics only as needed and considering the potential misuse of antibiotics may improve treatment outcomes in individuals who are scheduled to receive ICI treatment.

## Supplementary information

**Additional file 1. **Immune check point inhibitors, Treatment response in solid cancer. Non-evaluated, *N* = 24, ATB 60: antibiotics use within 60 days before ICI start, ATB 30: antibiotics use within 30 days before ICI start.

**Additional file 2.** Survival curves and the impact of antibiotics in solid cancer patients treated with ICIs. ATB 60: antibiotic use within 60 days prior to ICI treatment, ATB 30: antibiotic use within 30 days prior to ICI treatment.

**Additional file 3.** Multivariate analysis.

**Additional file 4.** Survival curves and the impact of antibiotics in NSCLC patients treated with ICIs. ATB 60: antibiotic use within 60 days prior to ICI treatment, ATB 30: antibiotic use within 30 days prior to ICI treatment.

**Additional file 5.** Survival curves and the impact of antibiotics administration in less than 7 days in solid cancer patients treated with ICI. ATB: antibiotics.

**Additional file 6.** Comparing between survival curves depending on ECOG and antibiotics. ATB: antibiotics, ECOG: Eastern Cooperative Oncology Group score.

## Data Availability

The data that support the findings of this study are available from the corresponding author but restrictions apply to the availability of these data, which were used under license for the current study, and so are not publicly available. Data are however available from the corresponding author upon reasonable request and with permission of the Institutional Review Board of the Seoul St. Mary’s Hospital.

## Abbreviations

BLBLI: Beta-lactam/beta-lactamase inhibitor
CD28: Cluster of differentiation 28
CI: Confidence interval
CR: Complete response
CTLA-4: Cytotoxic T-lymphocyte associated protein 4
ECOG PS: Eastern Cooperative Oncology Group Performance Status
EGFR: Epidermal growth factor receptor
FMT: Fecal microbiota transplantation
HR: Hazard ratio
ICI: Immune checkpoint inhibitor
IRB: Institutional Review Board
LCMV: Lymphocytic choriomeningitis virus
mAb: Monoclonal antibody
NSCLC: Non-small-cell lung carcinoma
OR: Odds ratio
PD: Progressive disease
PD-1: Programmed cell death protein-1
PD-L1: Programmed death-ligand 1
PR: Partial response
SD: Stable disease
