# Supplementary material for: The effect of antibiotics on the clinical outcomes of patients with solid cancers undergoing immune checkpoint inhibitor treatment: a retrospective study
Source: BMC Cancer. 2019 Nov 12;19:1100. doi: 10.1186/s12885-019-6267-z (PMC6852740; doi:10.1186/s12885-019-6267-z)
Supplement: Supplementary file 2 — Additional file 2. Survival curves and the impact of antibiotics in solid cancer patients treated with ICIs. ATB 60: antibiotic use within 60 days prior to ICI treatment, ATB 30: antibiotic use within 30 days prior to ICI treatment. [file 12885_2019_6267_MOESM2_ESM.pptx]

## Slide 1
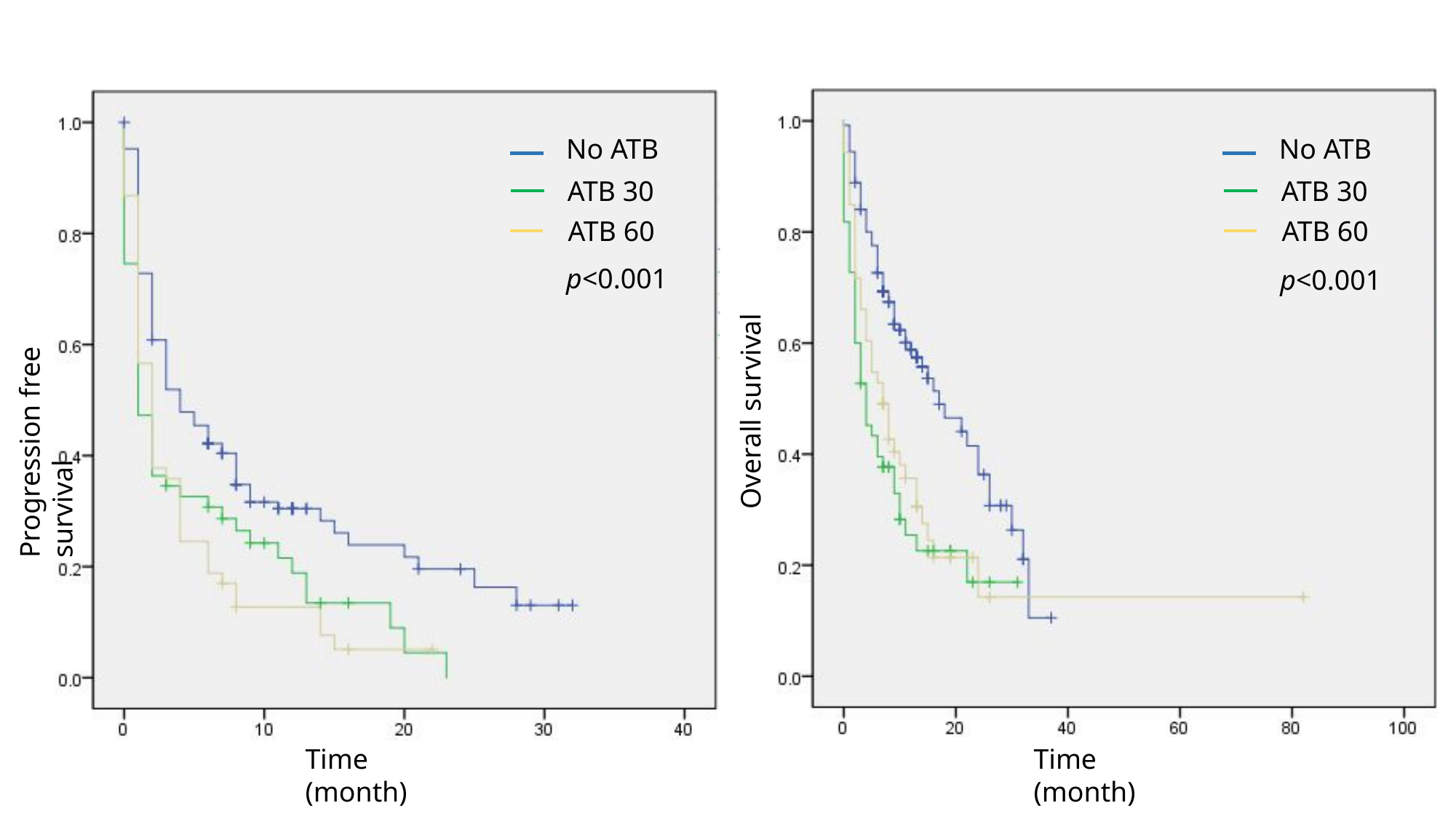

No ATB
No ATB
ATB 30
ATB 30
ATB 60
ATB 60
Progression free survival
p<0.001
p<0.001
Overall survival
Time (month)
Time (month)
