# Supplementary figures and images for: The effect of antibiotics on the clinical outcomes of patients with solid cancers undergoing immune checkpoint inhibitor treatment: a retrospective study
Source: BMC Cancer. 2019 Nov 12;19:1100. doi: 10.1186/s12885-019-6267-z (PMC6852740; doi:10.1186/s12885-019-6267-z)

## Slide 1
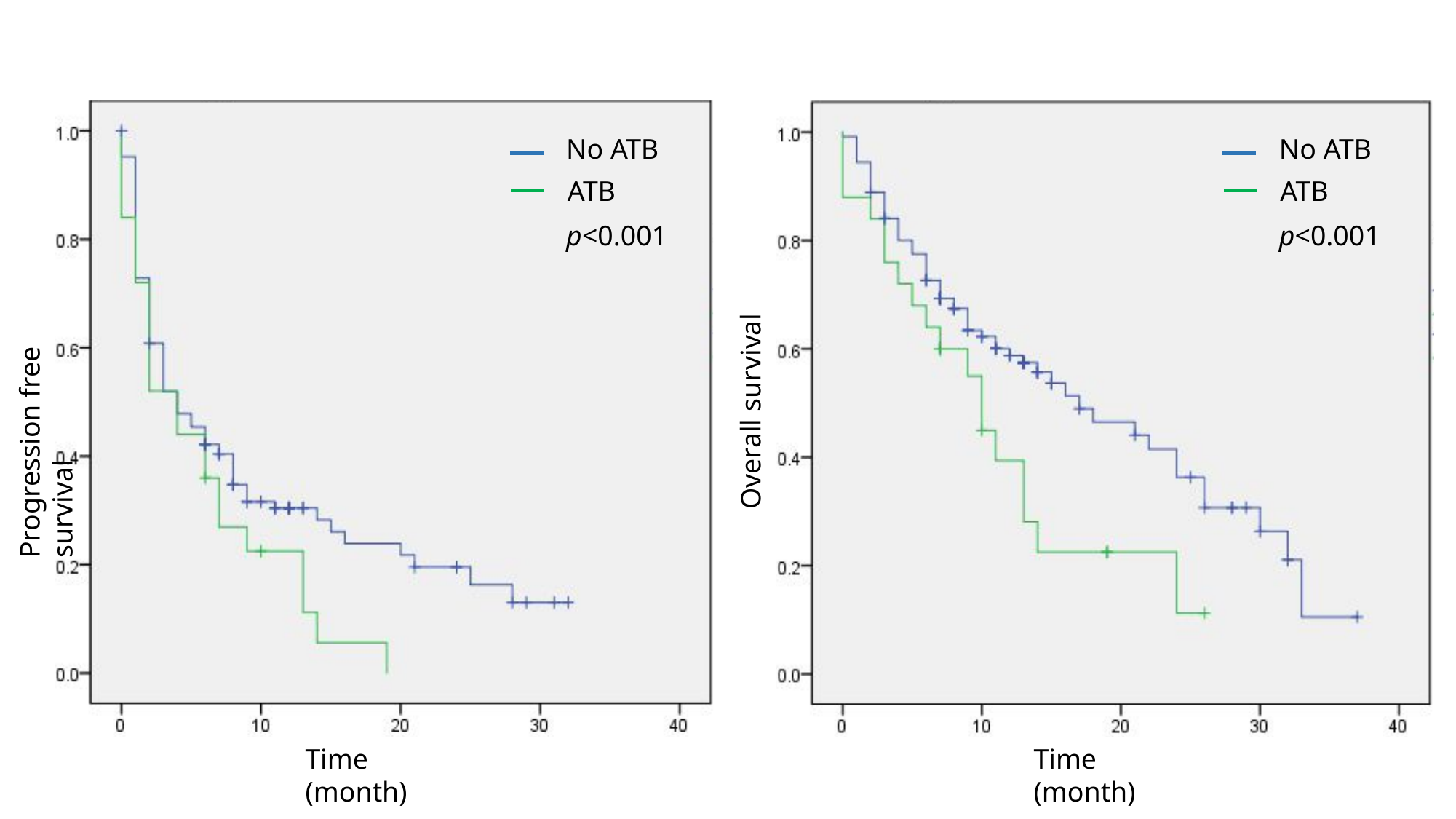

No ATB
No ATB
ATB
ATB
p<0.001
p<0.001
Progression free survival
Overall survival
Time (month)
Time (month)

Supplement: Supplementary file 5 — Additional file 5. Survival curves and the impact of antibiotics administration in less than 7 days in solid cancer patients treated with ICI. ATB: antibiotics. [file 12885_2019_6267_MOESM5_ESM.pptx]

## Slide 1
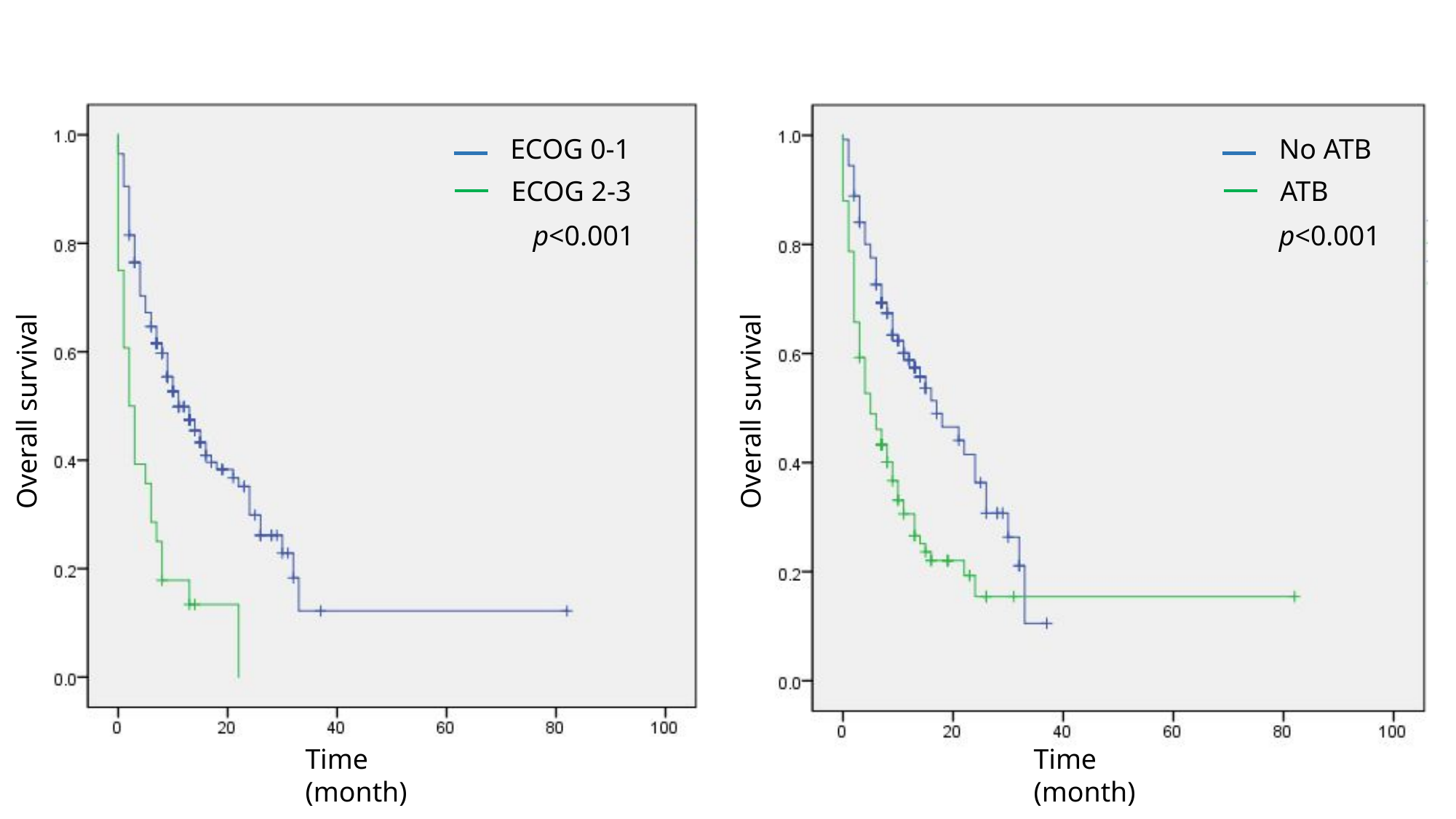

ECOG 0-1
No ATB
ECOG 2-3
ATB
p<0.001
p<0.001
Overall survival
Overall survival
Time (month)
Time (month)

Supplement: Supplementary file 6 — Additional file 6. Comparing between survival curves depending on ECOG and antibiotics. ATB: antibiotics, ECOG: Eastern Cooperative Oncology Group score. [file 12885_2019_6267_MOESM6_ESM.pptx]
